# Application of GOLD 2023 Initial Inhalation Therapy Recommendations in COPD patients: a real-world adherence and prognosis analysis

**DOI:** 10.7189/jogh.15.04324

**Published:** 2025-11-28

**Authors:** Dingding Deng, Dan Peng, Qing Song, Ling Lin, Cong Liu, Tao Li, Ping Zhang, Yuqin Zeng, Si Lei, Ping Chen

**Affiliations:** 1 Department of Respiratory and Critical Care Medicine, The First Affiliated Hospital of Shaoyang University, Shaoyang, Hunan, China; 2 Department of General Medicine, The Fourth People's Hospital of Nanchang County, Nanchang, Jiangxi, China; 3 Department of Pulmonary and Critical Care Medicine, the Second Xiangya Hospital, Central South University, Changsha, Hunan, China; 4 Research Unit of Respiratory Disease, Central South University, Changsha, Hunan, China; 5 Clinical Medical Research Center for Pulmonary and Critical Care Medicine, Hunan Province, China; 6 Diagnosis and Treatment Center of Respiratory Disease, Central South University, Changsha, Hunan, China; 7 Department of General Medicine, The Second Xiangya Hospital of Central South University, Changsha, Hunan, China

## Abstract

**Background:**

The Global Initiative for Chronic Obstructive Lung Disease (GOLD) 2023 report revised the combined chronic obstructive pulmonary disease (COPD) assessment. Patients were classified into groups A, B, and E, and the initial inhalation therapy recommendations were revised. This study aimed to investigate the application status of initial inhalation therapy recommendations in patients with COPD and determine whether adherence to the GOLD 2023 report could achieve a better prognosis.

**Methods:**

This was a prospective cohort study. Demographic data, COPD assessment test (CAT) and modified Medical Research Council (mMRC) scores, pulmonary function, GOLD grades, GOLD groups, number of exacerbations, comorbidities, and inhalation therapy were collected. The patients were classified into adherent and non-adherent groups based on the provision of initial inhalation therapy recommendations that aligned with the GOLD A, B, and E groupings. All patients finished one year of follow-up to collect data on the number of exacerbations and mortality.

**Results:**

A total of 1654 patients were enrolled, of whom 816 (49.3%) were in the adherent group. The patients in the adherent group had higher age, CAT and mMRC scores, and number of exacerbations and hospitalisations, higher proportion of combined with lung cancer and chronic heart disease, and worse pulmonary function. Patients in the adherent group had lower future exacerbations, frequent exacerbations, and hospitalisations. The patients in groups B and E who adhered to the GOLD 2023 report had lower future exacerbations, frequent exacerbations, and hospitalisations, while no significant difference was observed in group A (*P* < 0.05).

**Conclusions:**

In the real world, many patients with COPD do not receive the initial inhalation therapy recommended by the GOLD 2023 report. However, adherence to the GOLD 2023 report may decrease the risk of future exacerbation. It implied that improved the dissemination and uptake of GOLD 2023 recommendations is needed in the clinical practice.

Chronic obstructive pulmonary disease (COPD) is a chronic respiratory disease characterised by airflow limitation and is the third leading cause of death [1,2]. Therefore, precise treatment for patients with COPD is vital for improving their prognosis.

The Global Initiative for Chronic Obstructive Lung Disease (GOLD) programme provides recommendations for managing COPD and is revised annually. Until GOLD 2017, the combined COPD assessment tool classified patients into groups A, B, C, and D based on their COPD assessment test (CAT) or modified Medical Research Council (mMRC) scores and exacerbation or hospitalisation in the past year [3]. However, there has been a major revision, including a combined assessment tool and initial inhalation therapy recommendation, in the GOLD 2023 report. Patients are now classified into groups A, B, and E. The initial inhalation therapy recommended to patients in group A is a short- or long-acting bronchodilator. In contrast, group B receives a long-acting β2-agonist (LABA) + long-acting muscarinic antagonist (LAMA), while group E receives LABA + LAMA or LABA + LAMA + inhaled corticosteroid (ICS) [4]. Undeniably, this new grouping could simplify clinical assessment and management and optimise treatment recommendations for patients with COPD based on a randomised controlled trial. However, patients with COPD are complex and have complications in the real world [5]. Whether the initial inhalation therapy recommendations, according to the GOLD 2023 report, are appropriate for patients with COPD requires further investigation. In addition, it is unclear whether the clinicians will provide initial inhalation therapy to patients with COPD based on the GOLD 2023 report recommendation.

Therefore, this study aimed to investigate the application status of initial inhalation therapy recommendations in patients with COPD and determine whether adherence to the GOLD 2023 report would result in a better prognosis in the real world.

## METHODS

### Study participants

This was a multicentre prospective cohort study. All patients in this study were registered in The Current Status of Diagnosis and Treatment of COPD (RealDTC) (Register number: ChiCTR-POC-17010431) study between January 2023 and February 2024. The RealDTC study was launched to investigate the clinical characteristics, treatments, and prognosis of COPD in the Chinese population. The first patient recruited in the database (http://120.77.177.175:9007/a/login) was recorded on 1 December 2016 until now. A total of 13 hospitals including Hunan and Guangxi provinces participated in this study. The detail information had reported previously [6]. The patients were diagnosed with COPD according to the GOLD 2023 report: the ratio of forced expiratory volume in one second (FEV_1_) to forced vital capacity (FVC) was <0.70 after inhaling a bronchodilator [4]. We excluded patients with active tuberculosis, asthma, and severe heart, liver, or kidney disease.

This study was approved by the Institutional Review Board of the Second Xiangya Hospital of Central South University and conducted in accordance with the Declaration of Helsinki (2016076). Written informed consent was obtained from all patients.

### Data collection

Baseline data including age, sex, body mass index (BMI), education level, smoking (pack/y), smoking history, biofuel exposure, forced expiratory volume in one second predicted percentage (FEV_1%_pred), FEV_1_/FVC, GOLD grades, GOLD groups, CAT and mMRC scores, exacerbations and hospitalisations in the past year, inhalation therapy regimens, and comorbidities (including chronic heart disease, hypertension, lung cancer, bronchiectasis, and diabetes) were collected when patients first visited hospitals.

All patients completed one year of follow-up to collect data on exacerbations, frequent exacerbations, hospitalisations, and mortality.

### Study procedures

Patients were stratified into adherent and non-adherent groups based on their initial inhalation therapy regimens provided at their first hospitals visit. According to the GOLD 2023 report [4], the adherent group was defined as patients who received a short- or long-acting bronchodilator in group A, LABA + LAMA in group B, and LABA + LAMA or LABA + LAMA + ICS in group E.

### Variable definition

The CAT score consists of eight items including coughing, expectoration, chest tightness, sleep, energy, mood, exercise endurance, and the impact on daily activities, with scores ranging from 0 to 5, and the total scores ranging from 0 to 40. The mMRC score was used to evaluate dyspnoea, with scores ranging from 0 to 4. The higher the scores, the more severe the symptoms [7]. According to the GOLD 2023 report, patients were assigned to the three groups: group A, 0 to 1 exacerbation per year, no hospitalisation, CAT scores of <10, and mMRC scores of 0 to 1; group B, 0 to 1 exacerbation per year, no hospitalisation, CAT scores of ≥10, or mMRC scores of ≥2; and group E, exacerbations ≥2, or hospitalisation ≥1 per year [4].

A current smoker was defined as smoking ≥10 packs/y, and a former smoker was defined as smoking ≥10 packs/y but had not smoked for more than six months [8]. Exacerbation was defined as patients with COPD needing antibiotics, oral corticosteroids, or hospitalisation [9]. Frequent exacerbations were defined as ≥2 exacerbations per year [10]. In this study, all exacerbations and hospitalisations were caused by COPD. In order to better analyse the prognosis, the patients who adjust treatment, was defined as changes in the inhalation therapy drugs or stopped inhalation therapy drugs for more than three months during the one-year follow-up were excluded [11,12]. The biofuel exposure was defined as continuous exposure to biofuels for at least two hours a day for at least one year [13].

### Statistical analysis

Continuous variables with a normal distribution and homogeneity of variance are expressed as the mean ± standard deviation (SD); they were analysed using student *t* test. Otherwise, the variables are expressed as median and interquartile range (IQR) and were analysed using non-parametric tests. Categorical variables were analysed using the χ^2^ test. Odds ratios (OR) and 95% confidence intervals (95% CI) were calculated using a logistic regression. Statistical significance was set at *P* < 0.05. We used SPSS Statistics version 26.0 (IBM Corp., Armonk, USA) and Free Statistics software version 1.7.1 (Beijing Fengrui Technology Co., Ltd, Beijing, China) to conduct the statistical data analyses.

## RESULTS

### The clinical characteristics of the total COPD patients

**Figure 1:**
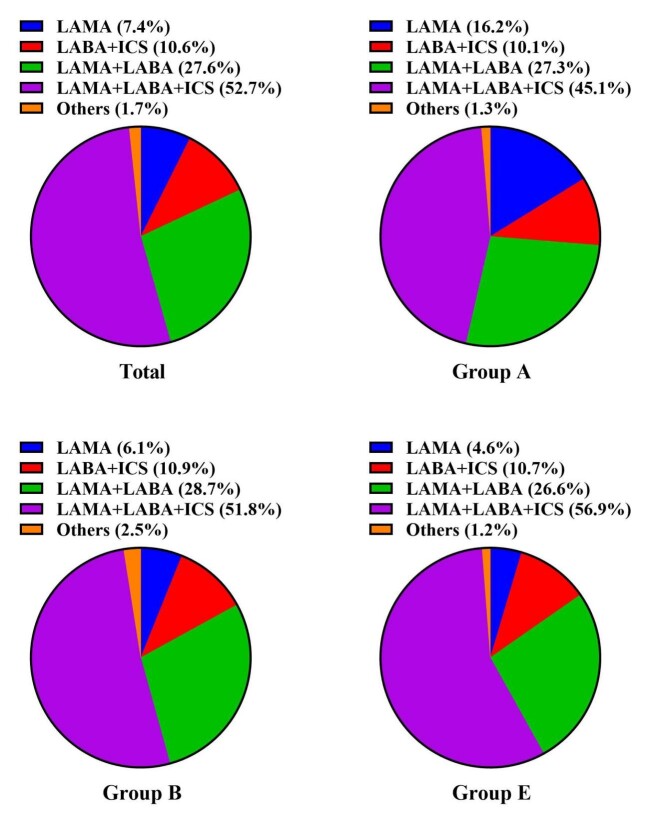
Proportion of inhalation therapy in different groups. Others including no inhalation therapy. ICS – inhaled corticosteroids, LAMA – long-acting muscarinic antagonist, LABA – long-acting β2-agonist.

A total of 1700 patients were enrolled, of whom 46 were excluded due to incomplete data. Finally, 1654 patients with COPD were enrolled in this study (Figure S1 in the **Online Supplementary Document**); 308 (18.6%) were classified into group A, 654 (39.5%) into group B, and 692 (41.9%) into group E. A total of 122 (7.4%) patients received LAMA, 456 (27.6%) received LABA + LAMA, 176 (10.6%) received LABA + ICS, and 872 (52.7%) received LABA + LAMA + ICS (**Table 1**). In group A, 16.2% of the patients received LAMA. In group B, 28.7% of the patients received LABA + LAMA. In group E, 83.5% of the patients received LABA + LAMA or LABA + LAMA + ICS (**Figure 1**).

**Table 1:** The baseline clinical characteristics of the total COPD patients

Variables	Total (n = 1654)
Age in years (x̄ ± SD)	65.0 ± 8.6
Sex, n (%)	
*Male*	1454 (87.9)
*Female*	200 (12.1)
BMI, kg/m^2^ (x̄ ± SD)	22.9 ± 3.5
Education level, n (%)	
*Junior high school and below*	1252 (75.7)
*High school and over*	402 (24.3)
Smoking history, n (%)	
*Never smoker*	354 (21.4)
*Former smoker*	566 (34.2)
*Current smoker*	734 (44.4)
Smoking, pack/y (MD, IQR)	40.0 (18.6, 53.0)
Biofuel exposure, n (%)	
*Yes*	375 (22.7)
*No*	1277 (77.3)
FEV_1%_pred, (x̄ ± SD)	58.4 ± 21.4
FEV_1_/FVC, (x̄ ± SD)	50.7 ± 12.1
GOLD grades, n (%)	
*1*	274 (16.6)
*2*	746 (45.1)
*3*	493 (29.8)
*4*	141 (8.5)
GOLD groups, n (%)	
*A*	308 (18.6)
*B*	654 (39.5)
*E*	692 (41.9)
CAT (x̄ ± SD)	13.7 ± 6.0
mMRC (MD, IQR)	2.0 (1.0, 2.0)
Exacerbations in the past year (MD, IQR)	1.0 (0.0, 2.0)
Hospitalisations in the past year (MD, IQR)	0.0 (0.0, 1.0)
Comorbidities, n (%)	
*Chronic heart disease*	118 (7.1)
*Hypertension*	189 (11.4)
*Lung cancer*	37 (2.2)
*Diabetes*	56 (3.4)
*Bronchiectasis*	97 (5.9)
Therapy, n (%)	
*LAMA*	122 (7.4)
*LABA+ICS*	176 (10.6)
*LAMA+LABA*	456 (27.6)
*LAMA+LABA+ICS*	872 (52.7)
Others*	28 (1.7)

BMI – body mass index, COPD – chronic obstructive pulmonary disease, CAT – COPD assessment test, FEV_1%_pred – forced expiratory volume in the first second predicted percentage, FVC – forced vital capacity, GOLD – Global Initiative for Chronic Obstructive Lung Disease, ICS – inhaled corticosteroids, IQR – interquartile range, LAMA – long-acting muscarinic antagonist, LABA – long-acting β2-agonist, MD – median, mMRC – modified Medical Research Council, SD – standard deviation, x̄ - mean

*Others included no inhalation therapy.

**Figure 2:**
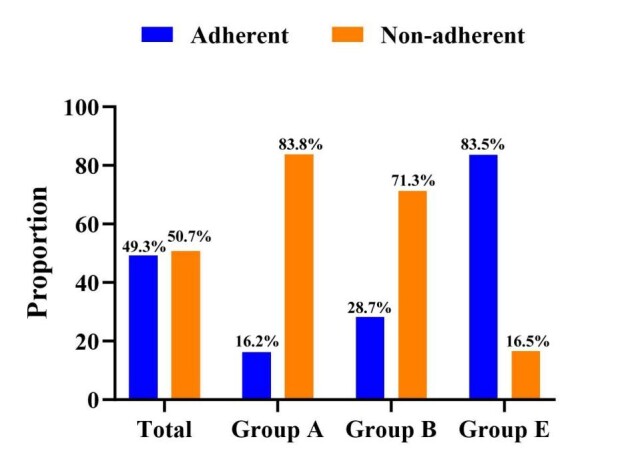
Proportion of non-adherent and adherent in the groups A, B, and E.

In all COPD patients, 49.3% of the patients adhered to the GOLD 2023 report that recommended inhalation therapy regimens. In group A, the proportion of adherence to the inhalation therapy regimen as recommended by the GOLD 2023 report was 16.2%. In group B, the proportion of adherence to the inhalation therapy regimens as recommended by the GOLD 2023 report was 28.7%. In group E, the proportion of adherence to the inhalation therapy regimen recommended by the GOLD 2023 report was 83.5% (**Figure 2**).

### The clinical characteristics of the patients who adhered to the GOLD 2023 report

The patients in the adherent group had higher age, CAT and mMRC scores, and number of exacerbations and hospitalisations. A higher proportion of these patients had lung cancer, chronic heart disease, and worse pulmonary function (*P* < 0.05) (**Table 2**).

**Table 2:** The clinical characteristics of the patients who adhered to the GOLD 2023 report

Variables	Non-adherent (n = 838)	Adherent (n = 816)	*P*-value
Age in years (x̄ ± SD)*	64.5 ± 8.6	65.5 ± 8.5	0.019§
Sex, n (%)†			0.580
*Male*	733 (87.5)	721 (88.4)	
*Female*	105 (12.5)	95 (11.6)	
BMI, kg/m^2^ (x̄ ± SD)*	23.1 ± 3.5	22.7 ± 3.5	0.024§
Education level, n (%)†			<0.001§
*Junior high school and below*	604 (72.1)	648 (79.4)	
*High school and over*	234 (27.9)	168 (20.6)	
Smoking history, n (%)†			0.289
*Never smoker*	189 (22.6)	165 (20.2)	
*Former smoker*	273 (32.6)	293 (35.9)	
*Current smoker*	376 (44.8)	358 (43.9)	
Smoking, pack/y (MD, IQR)‡	40.0 (15.0, 51.0)	40.0 (20.0, 55.0)	0.050
Biofuel exposure, n (%)†			0.196
*Yes*	179 (21.4)	196 (24.0)	
*No*	658 (78.6)	619 (76.0)	
FEV_1%_pred, (x̄ ± SD)*	60.0 ± 21.2	56.8 ± 21.6	0.002§
FEV_1_/FVC, (x̄ ± SD)*	51.5 ± 11.6	49.9 ± 12.6	0.008§
GOLD grades, n (%)†			0.002§
*1*	153 (18.3)	121 (14.8)	
*2*	392 (46.8)	354 (43.4)	
*3*	240 (28.6)	253 (31.0)	
*4*	53 (6.3)	88 (10.8)	
CAT (x̄ ± SD)*	12.4 ± 6.0	15.0 ± 5.8	<0.001§
mMRC, (MD, IQR)‡	1.0 (1.0, 2.0)	2.0 (1.0, 2.0)	<0.001§
Exacerbations in the past year, (MD, IQR)‡	0.0 (0.0, 1.0)	1.0 (1.0, 3.0)	<0.001§
Hospitalisations in the past year, (MD, IQR)‡	0.0 (0.0, 0.0)	1.0 (0.0, 1.0)	<0.001§
Comorbidities, n (%)†			
*Chronic heart disease*	46 (5.5)	72 (8.8)	0.008§
*Hypertension*	98 (11.7)	91 (11.2)	0.729
*Lung cancer*	11 (1.3)	26 (3.2)	0.010§
*Diabetes*	27 (3.2)	29 (3.6)	0.709
*Bronchiectasis*	42 (5.0)	55 (6.7)	0.135

BMI – body mass index, COPD – chronic obstructive pulmonary disease, CAT – COPD assessment test, FEV1%pred – forced expiratory volume in the first second predicted percentage, FVC – forced vital capacity, GOLD – Global Initiative for Chronic Obstructive Lung Disease, ICS – inhaled corticosteroids, IQR – interquartile range, LAMA – long-acting muscarinic antagonist, LABA – long-acting β2-agonist, MD – median, mMRC – modified Medical Research Council, SD – standard deviation, x̄ – mean

*Student *t* test.

†χ^2^ test.

‡Non-parametric tests.

§Statistically significant results.

Logistic regression analysis showed that the CAT scores (OR = 1.033; 95% CI = 1.013–1.053), exacerbations in the past year (OR = 1.405; 95% CI = 1.286–1.535), hospitalisations in the past year (OR = 2.519; 95% CI = 2.034–3.118), and combined with lung cancer (OR = 2.529; 95% CI = 1.156–5.530) were positively correlated with patients’ adherence to the GOLD 2023 report (*P* < 0.05) (**Table 3**).

**Table 3:** Multivariate logistic regression analysis of relative factors for patients who adhered to the GOLD 2023 report*

Variables	OR	95% CI	*P*-value†
Education level			
*Junior high school and below*	Reference		
*High school and over*	0.757	0.583, 0.983	0.036
CAT	1.033	1.013, 1.053	0.001
Exacerbations in the past year	1.405	1.286, 1.535	<0.001
Hospitalisations in the past year	2.519	2.034, 3.118	<0.001
Lung cancer			
*No*	Reference		
*Yes*	2.529	1.156, 5.530	0.020

BMI – body mass index, CAT – COPD assessment test, CI – confidence interval, COPD – chronic obstructive pulmonary disease, GOLD – Global Initiative for Chronic Obstructive Lung Disease, mMRC – modified Medical Research Council, OR – odds ratio

*Variables in the logistic regression model are age, BMI, education level, CAT, mMRC, GOLD grades, exacerbations in the past year, hospitalisations in the past year, lung cancer, and chronic heart disease.

†Statistically significant values.

### Future exacerbations and mortality of the patients who adhered to the GOLD 2023 report

During one year of follow-up, there were 106 patients lost follow-up and 239 patients adjust treatment were excluded. Finally, a total of 1309 patients were analysed the prognosis. Patients in the adherent group had lower future exacerbations, frequent exacerbations, and hospitalisations than those in the non-adherent group. However, there was no significant difference in mortality (Table S1–2 in the **Online Supplementary Document**).

In addition, in group A, there were no significant differences between the adherent and non-adherent groups in future exacerbations, frequent exacerbations, hospitalisations, and mortality (Table S3–4 in the **Online Supplementary Document**). In groups B and E, adherence to the GOLD 2023 report was associated with lower future exacerbations, frequent exacerbations, and hospitalisations (Table S5–8 in the **Online Supplementary Document**) (*P* < 0.05). In addition, there was no significant difference in mortality between the adherent and non-adherent patients in groups B and E.

## DISCUSSION

The GOLD report is the most widely accepted guideline to clinicians for managing and treating patients suffering from COPD. Combined COPD assessment is a vital tool for evaluating patients in GOLD reports, and it recommends the most appropriate initial inhalation therapy. Notably, there was a significant revision of the COPD combined assessment in the GOLD 2023 report, and groups C and D were merged into group E.

LABA + LAMA is the first choice for patients in group B, and LABA + LAMA or LABA + LAMA + ICS is the first choice for patients in group E [4]. Although a short- or long-acting bronchodilator is recommended for patients in group A based on the GOLD 2023 report, LAMA has been the most commonly used in the real world [14,15]. In fact, the clinicians did not strictly adhere to the GOLD 2023 report recommendations when providing initial inhalation therapy drugs for the COPD patients. LAMA, LABA + LAMA, LABA + ICS, or LABA + LAMA + ICS may be the first choice for patients in groups A, B, and E. In other words, not all patients are provided initial inhalation therapy according to the GOLD 2023 recommendations. In addition, considering the complexity of COPD in the real world, it is unclear whether the initial inhalation therapy recommendation based on the GOLD 2023 report can achieve a better prognosis.

In this prospective cohort study, we found that only 49.3% of patients with COPD were provided an inhalation therapy regimen in adherence to the GOLD 2023 report recommendation, meaning that more than half of the patients did not receive an initial inhalation therapy regimen consistent with the recommendations of the GOLD 2023 report. The proportion of patient adherence to the recommendations of the GOLD 2023 report was lowest in group A patients, followed by group B patients. The main reason for this was the excessive use of LABA + LAMA + ICS. This might be because in the real world, high-step inhalation therapy can better to alleviate patients' symptoms or the risk of exacerbation in the clinical practice. In addition, we found that the patients with a heavy burden of symptoms and a high risk of exacerbation were more likely to adhere to the GOLD 2023 report. This is foreseeable. After all, for the patients had a risk of exacerbation in the past year and heavy symptoms, adhering to the GOLD report recommendations can reduce the risk of future exacerbations. In addition, patients in groups B and E accounted for the majority, and these patients had a heavy symptom burden or a high risk of exacerbation. Therefore, a higher-step of initial inhalation therapy such as LABA + LAMA or LABA + LAMA + ICS was required. In fact, due to the variability of medical centres or health care system factors in China, especially for grassroots hospitals, adhering to the recommendations of the GOLD 2023 report has always been a rather challenging task. A previous study showed that there was a conflict between the GOLD 2011 and 2017 recommendations and clinical practice, mainly concerning overtreatment in GOLD groups A and B, and patients with a high symptom burden and high risk of exacerbation were more likely to be treated in conformity with the GOLD report [16]. This finding is consistent with our results. In addition, Alabi et al. [17] also found that groups A, B and C, but not group D, showed discordance in COPD treatment relative to the GOLD 2017 report.

Furthermore, we are the first to investigate whether adherence to the GOLD 2023 report would result in a better prognosis in the real world. We found that patient adherence to the GOLD 2023 report was associated with lower future exacerbations, frequent exacerbations, and hospitalisations. Jochmann et al. [18] found that GOLD report adherence did not seem to impact symptom prevalence, exacerbation rate, or pulmonary function decline after one year of follow-up, which is inconsistent with our study. This is related to the differences in the GOLD reports. This study aimed to investigate whether adherence to the GOLD 2023 report would result in a better prognosis following the huge revision in the combined COPD assessment and initial inhalation therapy recommendation.

Furthermore, subgroup analyses showed that adherence to the GOLD 2023 report was associated with lower future exacerbations, frequent exacerbations, and hospitalisations in groups B and E. A previous study confirmed that patients treated with LABA + LAMA or LABA + LAMA + ICS had a lower incidence and frequency of exacerbations than those treated with LAMA or LABA + ICS in groups B and E [19]. This finding is consistent with our results. However, adherence to the GOLD 2023 report did not achieve a better prognosis in group A patients. This implies that mono-LAMA was sufficient for the patients in group A. This could help to avoid excessive treatment. A previous study confirmed that the future exacerbations, frequent exacerbations, and mortality showed no differences between the different inhalation therapies in group A. In addition, there was no significant difference in mortality between the adherent and non-adherent groups. This discrepancy may be attributed to the relatively short follow-up duration and low mortality in the cohort. After all, previous studies had confirmed that the mortality during one year of follow-up was not high [11,19].

This study had several limitations. First, we did not explore whether adherence to the GOLD 2023 report could improve pulmonary function and symptoms in the patients. Second, one year follow-up period for analysing mortality might not be appropriate. Therefore, long-term of follow-up is needed in the future. Third, we did not consider the side effects of the treatment. However, this is a real-world study. We have included as many factors as possible that may affect prognosis. In addition, we did not include the variable of eosinophils. Only a small number of patients had their eosinophils examined in this study. Finally, we did not investigate the reasons why clinicians failed to provide patients with the relevant initial inhalation therapy as recommended by the GOLD 2023 report. This provides a direction for future research.

## CONCLUSIONS

In the real world, many patients with COPD do not receive the initial inhalation therapy recommended by the GOLD 2023 report. However, adherence to the GOLD 2023 report may decrease future exacerbation risk. It implied that improved the dissemination and uptake of GOLD 2023 recommendations is needed in the clinical practice.

## Additional material


Online Supplementary Document
