# Supplementary material for: Application of GOLD 2023 Initial Inhalation Therapy Recommendations in COPD patients: a real-world adherence and prognosis analysis
Source: J Glob Health. 2025 Nov 28;15:04324. doi: 10.7189/jogh.15.04324 (PMC12662025; doi:10.7189/jogh.15.04324)
Supplement: Online Supplementary Document [file jogh-15-04324-s001.pdf]

Supplement to: Deng D, Peng D, Song Q, Lin L, Liu C, Li T, Zhang P, Zeng Y, Lei S, Chen P. Application of GOLD 2023 Initial Inhalation Therapy Recommendations in COPD patients: a real-world adherence and prognosis analysis. J Glob Health. 2025;14:04324.

Table S1. Future exacerbations and mortality of the total patients who adhered to the GOLD 2023 report.

Variables	Total (n=1309)	Non-adherent (n=647)	Adherent (n=662)	*P*-value
Exacerbations during one year (median, IQR) [b]	0.0 (0.0, 0.0)	0.0 (0.0, 1.0)	0.0 (0.0, 0.0)	**<0.001**
Exacerbations, n (%) [c]				**<0.001**
No	1021 (79.2)	475 (74.2)	546 (84.1)	
Yes	268 (20.8)	165 (25.8)	103 (15.9)	
Frequent exacerbations, n (%) [c]				**<0.001**
No	1210 (93.9)	583 (91.1)	627 (96.6)	
Yes	79 (6.1)	57 (8.9)	22 (3.4)	
Hospitalizations during one year (median, IQR) [b]	0.0 (0.0, 0.0)	0.0 (0.0, 0.0)	0.0 (0.0, 0.0)	0.102
Hospitalizations, n (%) [c]				0.130
No	1168 (90.6)	572 (89.4)	596 (91.8)	
Yes	121 (9.4)	68 (10.6)	53 (8.2)	
Mortality, n (%) [c]	20 (1.5)	7 (1.1)	13 (2.0)	0.193

Notes: Bold *P*-values indicate statistical significance. [a] student *t* test; [b] non-parametric tests; [c] chi-squared test.
Abbreviations: GOLD, Global Initiative for Chronic Obstructive Lung Disease; IQR, interquartile range.

Table S2. Multivariate analysis for future exacerbations and mortality of the total COPD patients.

Variables	Non-adherent (n=647)	Adherent (n=662)	*P*-value
	OR (95% CI)		
Exacerbations	Reference	0.44 (0.32-0.61)	**<0.001**
Frequent exacerbations	Reference	0.29 (0.16-0.53)	**<0.001**
Hospitalizations	Reference	0.60 (0.38-0.96)	**0.031**
Mortality	Reference	1.11 (0.34-3.63)	0.858

Notes: Factors in the logistic regression model: Age, sex, BMI, smoking history, biofuel exposure, FEV1%, FEV1/FVC, CAT, mMRC, comorbidities, and exacerbations in the past year. The bold *P*-values indicate statistical significance.

Abbreviations: BMI, body mass index; COPD, chronic obstructive pulmonary disease; CAT, COPD Assessment Test; FEV_1 %pred, forced expiratory volume in the first second predicted percentage; FVC, forced vital capacity; mMRC, modified Medical Research Council; OR, odds ratio; 95% CI, 95% confidence interval.

Table S3. Future exacerbations and mortality of the group A patients who adhered to the GOLD 2023 report.

Variables	Total (n=233)	Non-adherent (n=208)	Adherent (n=25)	*P*-value
Exacerbations during one year (median, IQR) [b]	0.0 (0.0, 0.0)	0.0 (0.0, 0.0)	0.0 (0.0, 0.0)	0.891
Exacerbations, n (%) [c]				1.000
No	192 (83.8)	171 (83.8)	21 (84.0)	
Yes	37 (16.2)	33 (16.2)	4 (16.0)	
Frequent exacerbations, n (%) [c]				0.702
No	210 (91.7)	186 (91.2)	24 (96.0)	
Yes	19 (8.3)	18 (8.8)	1 (4.0)	
Hospitalizations during one year (median, IQR) [b]	0.0 (0.0, 0.0)	0.0 (0.0, 0.0)	0.0 (0.0, 0.0)	0.52
Hospitalizations, n (%) [c]				0.626
No	217 (94.8)	194 (95.1)	23 (92.0)	
Yes	12 (5.2)	10 (4.9)	2 (8.0)	
Mortality, n (%) [c]	4 (1.7)	4 (1.9)	0 (0.0)	1.000

Notes: [a] student *t* test; [b] non-parametric tests; [c] chi-squared test.

Abbreviations: GOLD, Global Initiative for Chronic Obstructive Lung Disease; IQR, interquartile range.

Table S4. Multivariate analysis for future exacerbations and mortality of the group A patients.

Variables	Non-adherent (n=208)	Adherent (n=25)	*P*-value
	OR (95% CI)		
Exacerbations	Reference	1.49 (0.41-5.38)	0.547
Frequent exacerbations	Reference	0.58 (0.06-6.20)	0.656
Hospitalizations	Reference	1.91 (0.32-11.45)	0.478
Mortality	N/A	N/A	N/A

Notes: Factors in the logistic regression model: Age, sex, BMI, smoking history, biofuel exposure, FEV1%, FEV1/FVC, CAT, mMRC, comorbidities, and exacerbations in the past year.

Abbreviations: BMI, body mass index; COPD, chronic obstructive pulmonary disease; CAT, COPD Assessment Test; FEV_1 %pred, forced expiratory volume in the first second predicted percentage; FVC, forced vital capacity; mMRC, modified Medical Research Council; OR, odds ratio; 95% CI, 95% confidence interval

Table S5. Future exacerbations and mortality of the group B patients who adhered to the GOLD 2023 report.

Variables	Total (n=532)	Non-adherent (n=382)	Adherent (n=150)	*P*-value
Exacerbations during one year (median, IQR) [b]	0.0 (0.0, 0.0)	0.0 (0.0, 1.0)	0.0 (0.0, 0.0)	**<0.001**
Exacerbations, n (%) [c]				**<0.001**
No	404 (76.7)	269 (71.0)	135 (91.2)	
Yes	123 (23.3)	110 (29.0)	13 (8.8)	
Frequent exacerbations, n (%) [c]				**0.010**
No	493 (93.5)	348 (91.8)	145 (98.0)	
Yes	34 (6.5)	31 (8.2)	3 (2.0)	
Hospitalizations during one year (median, IQR) [b]	0.0 (0.0, 0.0)	0.0 (0.0, 0.0)	0.0 (0.0, 0.0)	**0.002**
Hospitalizations, n (%) [c]				**0.003**
No	477 (90.5)	334 (88.1)	143 (96.6)	
Yes	50 (9.5)	45 (11.9)	5 (3.4)	
Mortality, n (%) [c]	5 (0.9)	3 (0.8)	2 (1.3)	0.624

Notes: Bold *P*-values indicate statistical significance. [a] student *t* test; [b] non-parametric tests; [c] chi-squared test.

Abbreviations: GOLD, Global Initiative for Chronic Obstructive Lung Disease; IQR, interquartile range.

Table S6. Future exacerbations and mortality of the group E patients who adhered to the GOLD 2023 report.

Variables	Total (n=544)	Non-adherent (n=54)	Adherent (n=487)	*P*-value
Exacerbations during one year (median, IQR) [b]	0.0 (0.0, 0.0)	0.0 (0.0, 1.0)	0.0 (0.0, 0.0)	**<0.001**
Exacerbations, n (%) [c]				**<0.001**
No	425 (79.7)	35 (61.4)	390 (81.9)	
Yes	108 (20.3)	22 (38.6)	86 (18.1)	
Frequent exacerbations, n (%) [c]				**0.004**
No	507 (95.1)	49 (86.0)	458 (96.2)	
Yes	26 (4.9)	8 (14.0)	18 (3.8)	
Hospitalizations during one year (median, IQR) [b]	0.0 (0.0, 0.0)	0.0 (0.0, 0.0)	0.0 (0.0, 0.0)	**0.002**
Hospitalizations, n (%) [c]				**0.003**
No	474 (88.9)	44 (77.2)	430 (90.3)	
Yes	59 (11.1)	13 (22.8)	46 (9.7)	
Mortality, n (%) [c]	11 (2.0)	0 (0.0)	11 (2.3)	0.616

Notes: Bold *P*-values indicate statistical significance. [a] student *t* test; [b] non-parametric tests; [c] chi-squared test.

Abbreviations: GOLD, Global Initiative for Chronic Obstructive Lung Disease; IQR, interquartile range.

Table S7. Multivariate analysis for future exacerbations and mortality of the group B patients.

Variables	Non-adherent (n=382)	Adherent (n=150)	*P*-value
	OR (95% CI)		
Exacerbations	Reference	0.21 (0.11-0.39)	**<0.001**
Frequent exacerbations	Reference	0.27 (0.08-0.91)	**0.034**
Hospitalizations	Reference	0.30 (0.11-0.79)	**0.014**
Mortality	Reference	1.70 (0.23-12.86)	0.606

Notes: Factors in the logistic regression model: Age, sex, BMI, smoking history, biofuel exposure, FEV1%, FEV1/FVC, CAT, mMRC, comorbidities, and exacerbations in the past year. The bold *P*-values indicate statistical significance.

Abbreviations: BMI, body mass index; COPD, chronic obstructive pulmonary disease; CAT, COPD Assessment Test; FEV_1 %pred, forced expiratory volume in the first second predicted percentage; FVC, forced vital capacity; mMRC, modified Medical Research Council; OR, odds ratio; 95% CI, 95% confidence interval.

Table S8. Multivariate analysis for future exacerbations and mortality of the group E patients.

**Variables**	**Non-adherent (n=54)**	**Adherent (n=487)**	***P*-value**
	**OR (95% CI)**		
Exacerbations	Reference	0.31 (0.17-0.59)	**<0.001**
Frequent exacerbations	Reference	0.16 (0.06-0.41)	**<0.001**
Hospitalizations	Reference	0.27 (0.12-0.60)	**0.001**
Mortality	N/A	N/A	N/A

Notes: Factors in the logistic regression model: Age, sex, BMI, smoking history, biofuel exposure, FEV1%, FEV1/FVC, CAT, mMRC, comorbidities, and exacerbations in the past year. The bold *P*-values indicate statistical significance.

Abbreviations: BMI, body mass index; COPD, chronic obstructive pulmonary disease; CAT, COPD Assessment Test; FEV_1 %pred, forced expiratory volume in the first second predicted percentage; FVC, forced vital capacity; mMRC, modified Medical Research Council; OR, odds ratio; 95% CI, 95% confidence interval.

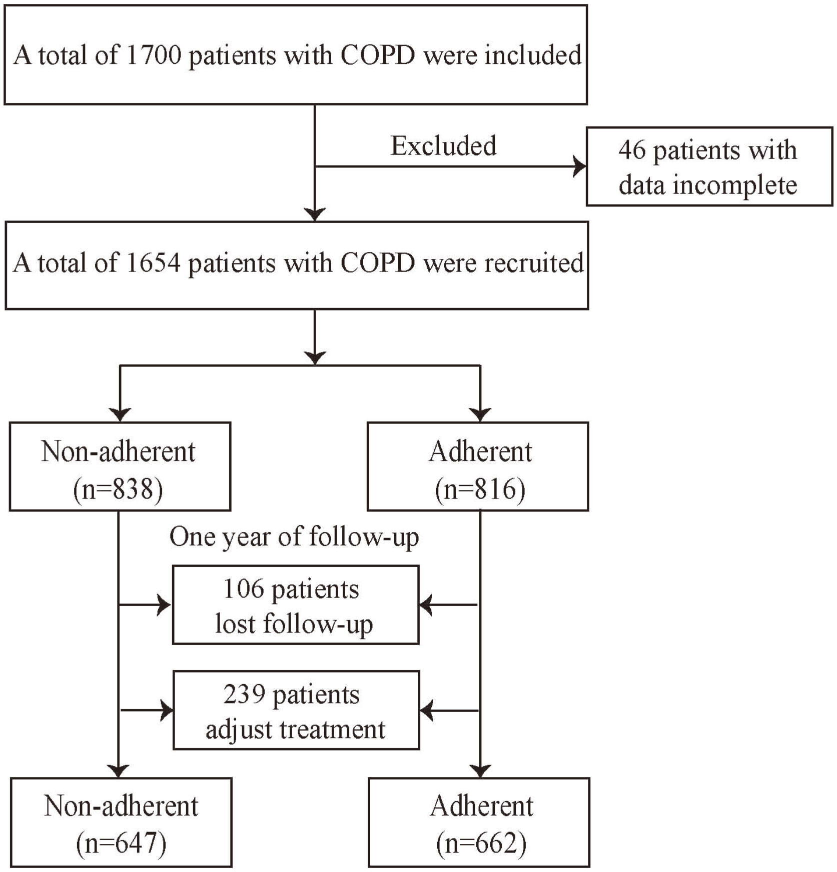

Figure S1. Flow chart of this study. COPD, chronic obstructive pulmonary disease.
